# Navigating the human-monkeypox virus interactome: HuPoxNET atlas reveals functional insights

**DOI:** 10.3389/fmicb.2024.1399555

**Published:** 2024-08-02

**Authors:** Raghav Kataria, Naveen Duhan, Rakesh Kaundal

**Affiliations:** ^1^Department of Plants, Soils, and Climate, College of Agriculture and Applied Sciences, Logan, UT, United States; ^2^Bioinformatics Facility, Center for Integrated BioSystems, Logan, UT, United States; ^3^Department of Computer Science, College of Science, Utah State University, Logan, UT, United States

**Keywords:** HuPoxNET, monkeypox, computational algorithms, protein–protein interactions, drug targets, tecovirimat, M11L, F1L

## Abstract

Monkeypox virus, a close relative of variola virus, has significantly increased the incidence of monkeypox disease in humans, with several clinical symptoms. The sporadic spread of the disease outbreaks has resulted in the need for a comprehensive understanding of the molecular mechanisms underlying disease infection and potential therapeutic targets. Protein–protein interactions play a crucial role in various cellular processes and regulate different immune signals during virus infection. Computational algorithms have gained high significance in the prediction of potential protein interaction pairs. Here, we developed a comprehensive database called HuPoxNET (https://kaabil.net/hupoxnet/) using the state-of-the-art MERN stack technology. The database leverages two sequence-based computational models to predict strain-specific protein–protein interactions between human and monkeypox virus proteins. Furthermore, various protein annotations of the human and viral proteins such as gene ontology, KEGG pathways, subcellular localization, protein domains, and novel drug targets identified from our study are also available on the database. HuPoxNET is a user-friendly platform for the scientific community to gain more insights into the monkeypox disease infection and aid in the development of therapeutic drugs against the disease.

## 1 Introduction

In the past decade, infectious diseases caused by various virus families (coronavirus, orthopoxvirus, myxovirus, alphavirus, etc.) continue to pose a significant threat to global public health, necessitating comprehensive efforts to understand the molecular complexities of their pathogenesis (Meganck and Baric, 2021). Among these pathogens, monkeypox virus (MPXV), belonging to *Poxviridae* family, has recently emerged as a notable concern due to its potential to cause outbreaks in humans, with reported cases spanning multiple continents. Monkeypox virus, closely related to variola virus (the causative agent of smallpox), causes monkeypox (abbreviated as “mpox”) disease in humans and displays similar clinical symptoms, although with generally milder outcomes. The zoonotic nature of the disease highlights the critical importance of comprehending its molecular mechanisms of infection, host-virus infection, and potential for cross-species transmission (Alakunle et al., 2020; Pastula and Tyler, 2022). Despite being a rare disease, the sporadic outbreaks of monkeypox have prompted concerns within the global health community (Li et al., 2022). The Center for Disease Control and Prevention (CDC) has reported cases of MPXV infection (91,328 cases globally and 31,010 cases in the United States as of October 26, 2023),^1^ resulting in fatalities, and emphasizing the need for a comprehensive understanding of pathogenic mechanisms of the virus and potential treatment options. Monkeypox infections in humans can result in a several clinical symptoms, including headache, fatigue, low-grade fever, lethargy, and others (McCollum and Damon, 2013; Cheema et al., 2022). Similar to other orthopoxviruses, such as vaccinia virus and variola virus, MPXV enters host cells through a complex of interactions between viral envelope proteins and host cell receptors. Once inside the host cell, the virus hijacks the cellular machinery to replicate, assemble, and ultimately release progeny virions (Aljabali et al., 2022).

Monkeypox virus emerged within the orthopoxvirus and evolved into various genetic variants over time. The virus has been divided into three clades—Congo Basin (clade 1), West African (clade 2), and current outbreaks outside Africa (clade 3) (Babkin et al., 2022; Luna et al., 2022). These distinctions are based on the mutations in coding regions involved in host recognition (Berthet et al., 2021). Clade 3 has diversified into various lineages, with A.1 and A.2 diverging from hMPXV-1A. Furthermore, lineage B.1 is considered to be associated to current outbreak of the disease and has undergone micro-evolution, resulting into several clusters such as B1.1, B1.2, B1.3, B1.4 etc. (Chakraborty et al., 2022; Isidro et al., 2022). This ongoing viral evolution suggests adaptability to new hosts and environments.

Various vaccines and drugs have been approved against the monkeypox disease. It has been reported that the smallpox vaccine offers 85% cross-protection against MPXV (Reynolds and Damon, 2012). The Center of Disease Control and Prevention (CDC) recommended the ACAM2000™ vaccine that only reduced symptoms without preventing the disease and had unknown side effects (Alakunle et al., 2020). JYNNEOS (IMVAMUNE) vaccine was authorized by European Medicines Agency (EMA) and Food and Drug Administration (FDA) for intradermal injection in smaller doses, thus increasing treatment accessibility (Li et al., 2022). LC16m8, produced in cell culture, has a higher safety profile and no serious side effects (Kennedy et al., 2011). FDA-approved antiviral drugs such as brincidofovir, tecovirimat, cidofovir, etc. are also available for monkeypox disease treatment (Rizk et al., 2022). However, their effectiveness against the current outbreak needs reevaluation.

Protein–protein interactions (PPIs) play a pivotal role in various molecular processes. During the initial stages of infection, viral proteins interact with host cell proteins through a myriad of interactions, which involve receptor recognition, endocytosis, and fusion events that facilitate viral entry (Saghazadeh and Rezaei, 2022). Subsequently, DNA replication of virus occurs in cytoplasmic structures known as factories (originally called Guarnieri bodies), which then helps in the formation of immature and mature virions that are responsible for infection (Kaler et al., 2022). As infection progresses, the virus employs various mechanisms to subvert host cellular processes for viral replication and protein synthesis, followed by modulation of signaling pathways, and inhibition of host defense mechanisms. Elucidation of the potential PPIs can provide valuable insights into the infection mechanisms and subsequent host defense responses, thus enabling the identification of potential therapeutic targets (Chadha et al., 2022; Kataria et al., 2023).

Amidst the era of bioinformatics and big data, the advancement in computational algorithms has revolutionized our ability to predict and analyze PPIs, enabling the researchers to decipher the complex network of molecular interactions involved in pathogenic infection. Various computational models have been developed for the prediction of host-pathogen PPIs, which offer a systematic approach for the identification of candidate interaction pairs between host and pathogen proteins (Skrabanek et al., 2008). Among the available computational models, the sequence-based algorithms (interolog and domain-based) have gained high popularity to predict PPIs between host and pathogen proteins. The sequence homology-based interolog approach exploits the principle of evolutionary conservation of PPIs across species, allowing the transfer of known interactions from model organisms to less-characterized pathosystems (Matthews et al., 2001). On the other hand, domain-based prediction focuses on identifying significant functional domains within proteins that mediate interactions between species (Li et al., 2019).

To aggregate and disseminate the wealth of protein interaction-related information and functional annotations, the development of a specialized database is necessary. In line with this, we aim to develop the database “HuPoxNET” that will serve as a centralized repository, consolidating curated data on Human-MPXV PPIs, and functional annotations of the predicted interactions. By integrating diverse sources of information, the database will provide scientific community with a comprehensive platform to explore, analyze, and understand the underlying molecular mechanisms of MPXV infection. HuPoxNET is available for public use at: https://kaabil.net/hupoxnet/.

## 2 Materials and methods

### 2.1 Design and implementation

**Figure 1:**
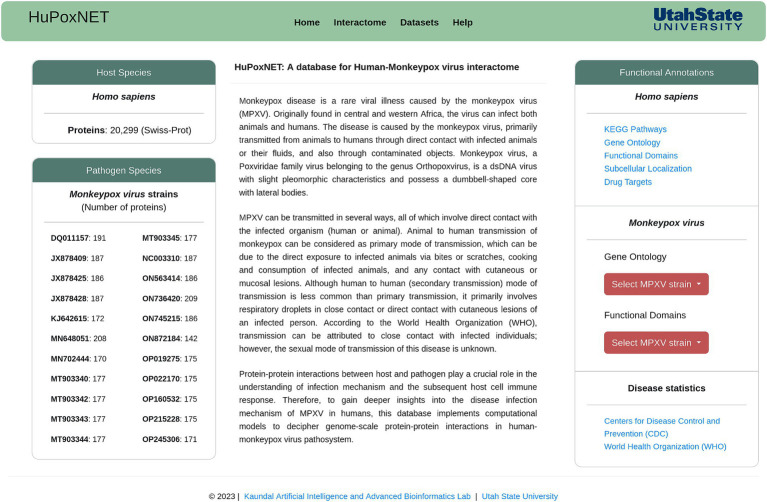
Homepage of HuPoxNET, representing monkeypox virus strains, various protein annotations, and functionalities available on the database.

For the development of a robust and dynamic database, we employed state-of-the-art MERN stack, which comprises of four essential components: (i) MongoDB, (ii) Express.js, (iii) React, and (iv) Node.js. This combination of technologies is flexible, scalable, and highly suitable for web applications. MongoDB serves as a database management system for storing the large amount of biological data. Express.js (backend framework) was utilized to create RESTful APIs and handle hypertext transfer protocol (HTTP) requests. React, a JavaScript library, was implemented to create a user-friendly interface that facilitates an interactive and responsive platform to access the available data and for protein interaction network visualization. Node.js forms the runtime environment for the server, thus ensuring efficient communication between frontend and backend for high-speed data retrieval and processing. The ‘Homepage’ of the database is presented in Figure 1.

### 2.2 HuPoxNET datasets

The database consists of 22 MPXV strains interacting with human proteins. Publicly available databases and specialized resources were leveraged for protein datasets collection. The proteome (20,299 proteins) of human was obtained from UniProt Swiss-Prot.^2^ On the other hand, the proteins of MPXV strains were obtained from NCBI.^3^ To reduce the sequence redundancy, CD-HIT (Fu et al., 2012) at 100% similarity was implemented on all the downloaded proteomes. The users can access these protein datasets through the “Datasets” page on the database, which will direct them to the actual data source.

Being comprehensive in nature, HuPoxNET is a resource for various functional annotations of the host and pathogen proteins. These include Kyoto Encyclopedia of Genes and Genomes (KEGG) pathways, gene ontology (GO), functional domains, subcellular localization, and potential drug targets. GO terms and KEGG pathways for human proteins were obtained from Ensembl.^4^ For functional domains, we analyzed the proteins with InterProScan (Jones et al., 2014). The subcellular localization information of human proteins was obtained from the Human Protein Atlas^5^ (Thul and Lindskog, 2018). The database also contains the information about the potential drugs against MPXV infection obtained from DrugBank.^6^

### 2.3 Computational models for human-MPXV Interactome

To implement the sequence-based computational models (interolog and domain) for the prediction of human-MPXV PPIs, we utilized the available gold standard protein–protein interaction databases (BioGRID, DIP, HPIDB, IntAct, MINT, and VirHostNet) and domain-domain interaction (DDI) databases (3did, DOMINE, and IDDI). These databases were extensively filtered to extract the experimentally validated and species-specific (here, human-virus) interactions, while the non-validated interactions were simultaneously discarded. This step was performed to reduce the false positive predictions. In homology-based interolog model, the protein datasets were subjected to BLAST search against the above-mentioned standard databases to predict the interactions. On the other hand, in domain-based model, we employed HMMER (Eddy, 2011) against Pfam as reference database to identify significant domains of the host and pathogen proteins to predict the potential PPIs. Furthermore, Cytoscape.js (Shannon et al., 1971) plugin was employed for enhanced network visualization of the predicted interactome.

## 3 Results and discussion

The understanding of protein interaction network is essential to gain insights into the biological and molecular processes that regulate various immune responses in a cellular environment. Several studies have reported the role of PPIs in the determination of significant protein hubs and molecular pathways that are involved in defense responses during the biotic stresses (Dhillon et al., 2020; Kataria and Kaundal, 2022). HuPoxNET is a comprehensive platform for the scientific community, specific to human disease-related research. The database consists of different functional annotations of the human and viral proteins, and protein interaction prediction tool with enhanced network visualization.

### 3.1 Interactome module

**Figure 2:**
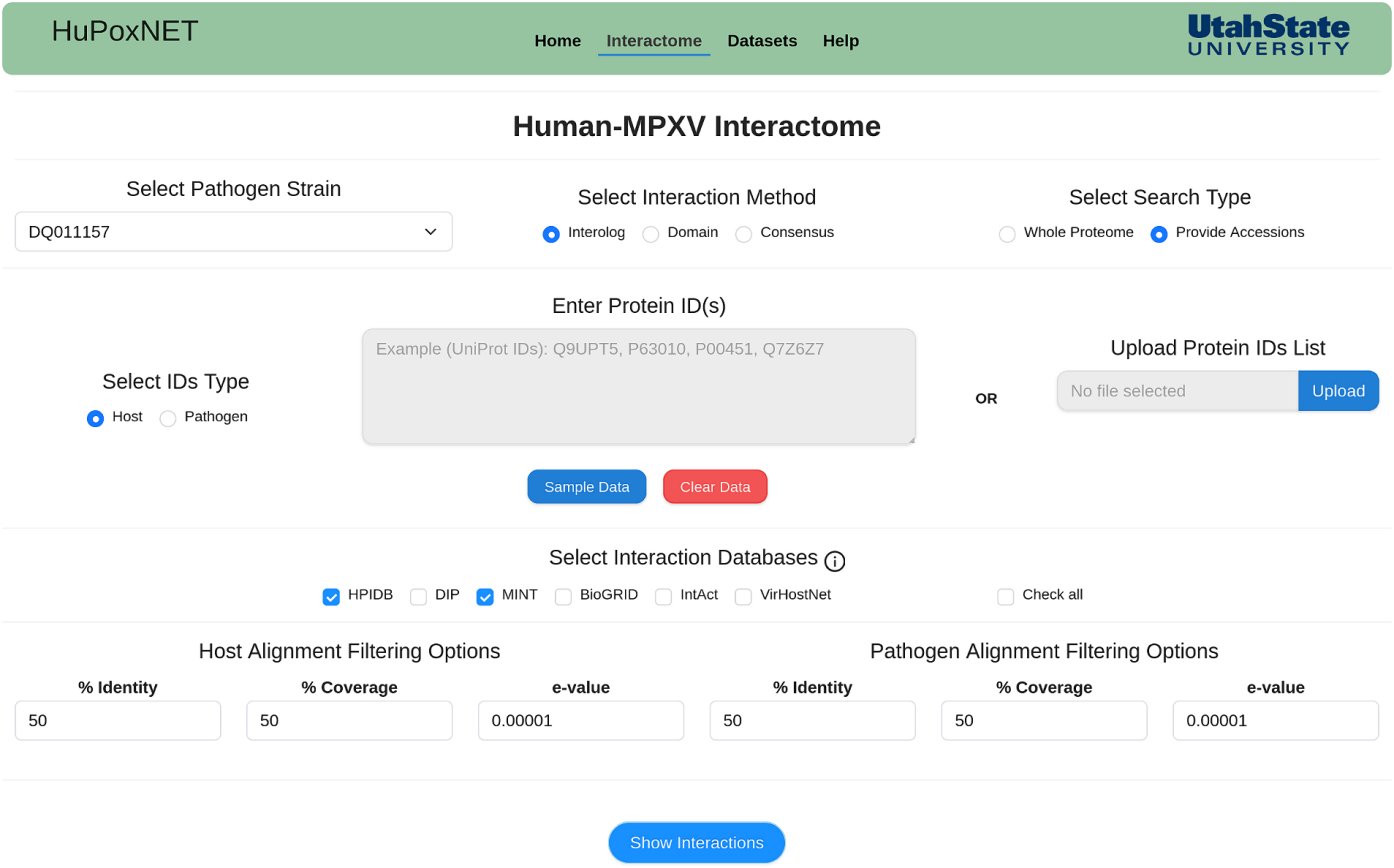
Interactome module of HuPoxNET. The users can select the MPXV strain of interest, select interaction method, and predict interactions based on whole proteome or proteins of their choice.

Monkeypox disease is caused in humans by different MPXV strains that differ in their evolutionary patterns (Kataria et al., 2023). In line with this, we implemented 22 MPXV strains causing infection in humans. HuPoxNET supports two sequence-based computational models (interolog and domain) for the prediction of PPIs between human and MPXV proteins. These models are integrated in the “Interactome” module of the database (Figure 2), which filters the precalculated interactions based on various parameters selected by the users. In this module, the users can predict the PPIs using the desired model (interolog, domain, or consensus). The users can select the interaction model of choice, followed by the selection of the available standard PPI and/or DDI databases, and subsequently change the BLAST alignment parameters (identity, coverage, and *e*-value) for both host and pathogen. The “consensus” model allows the user to predict the interactions based on both the interolog and domain model. This model outputs the common interactions predicted by both computational models, thus increasing the reliability of the predicted interactome.

**Figure 3:**
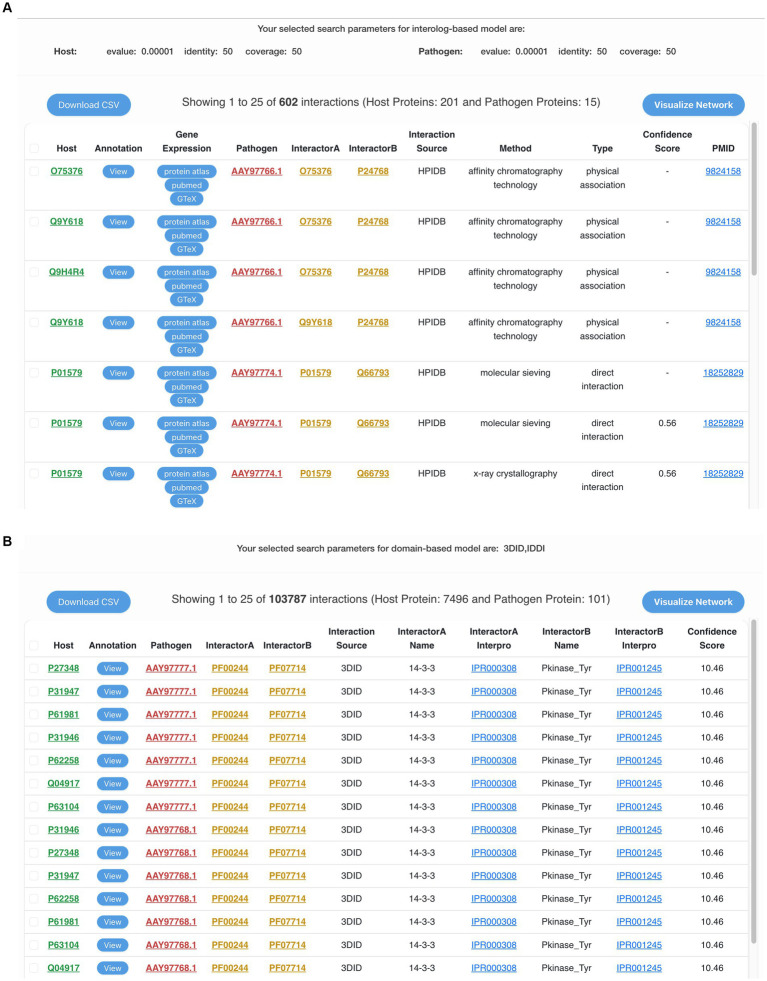
**(A)** The output of interolog-based search on the interactome module of HuPoxNET. Information such as predicted interacted pair, gene expression of host proteins, interaction method, confidence score, and NCBI PubMed ID is displayed. **(B)** The output of domain-based search on the interactome module of HuPoxNET. Information such as protein interaction predictions, standard interaction database source, domain names, interpro IDs, and confidence score is displayed.

Alternatively, instead of using the whole proteome, the users can also enter the protein IDs or provide a list of protein IDs of either human or MPXV proteins. This will filter the interactions based on the given protein IDs, thus making the prediction specific and time-efficient. Based on the selected MPXV strain, the resulting table consists of the potential interactions between human and MPXV proteins, corresponding interaction pair from the selected standard database(s), experimental method, domain name, InterProScan ID, source database name, PubMed ID, and confidence score (Figures 3A,B). Additionally, the human proteins in the predicted interactions have been linked to PubMed, Human Protein Atlas, and GTEx portal to access gene expression and related data to monkeypox disease, thus enriching the database with various functional annotations. To enhance user experience and provide more protein information, the resulting proteins in the interactions are also externally linked to the respective publicly available biological databases such as UniProt, NCBI, etc. The predicted interactions can also be downloaded in a comma-separated values (csv) file format. To make the search more user-friendly, each human protein in the predicted interactions is linked to its protein annotations from the results page of interactome module. This comprehensive annotation page consists of various functional annotations of proteins such as gene ontology (GO) terms, KEGG pathways, drug targets, etc.

**Figure 4:**
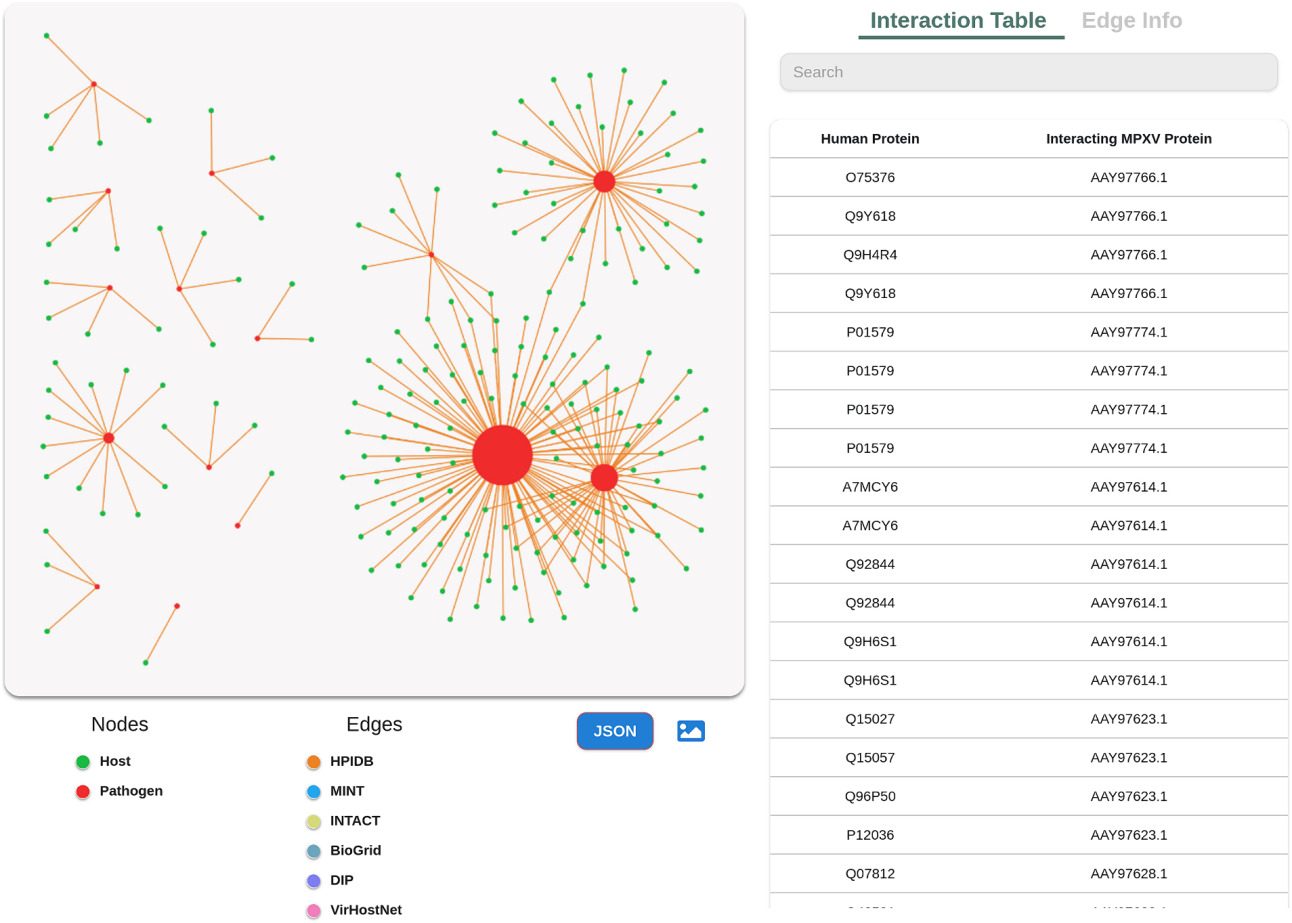
Network visualization of the predicted interactome. Green color in the network represents host protein and red represents pathogen protein. The edges with various colors represent different standard protein interaction databases. Size of each protein node is associated with degree of the protein.

Following the predictions, the user can visualize the predicted interactome, which is implemented using Cytoscape. The network consists of the predicted interactions, whereby the nodes are sized based on the degree of the protein. Higher the protein degree, larger is the node size, and vice versa. The colored edges in the network corresponds to the different gold standard databases selected by the user to predict the interactions (Figure 4). Selecting a node in the network provides the user with more information such as degree of the protein, which is further linked to external resources. The users can also modify the network according to their research question, and download it in PNG format. Additionally, the database allows the user to download the network in JSON format, thus enabling the users to import the network in advanced visualization tools and perform various analysis to extract more useful information.

### 3.2 Protein annotations

The understanding of the molecular mechanisms underlying disease infection and subsequent host defense responses rely on the functional annotations of the proteins involved in the interactions (Hong et al., 2019). HuPoxNET serves as the hub of functional annotations of the human and MPXV proteins involved in the interactions. Various computational tools have been developed, which were implemented to predict the different annotations of the interacting proteins in monkeypox disease. These annotations include functional domains of the proteins, GO terms, KEGG pathways, protein subcellular localization, and drug targets, which can be accessed from the ‘Homepage’ of the database.

**Figure 5:**
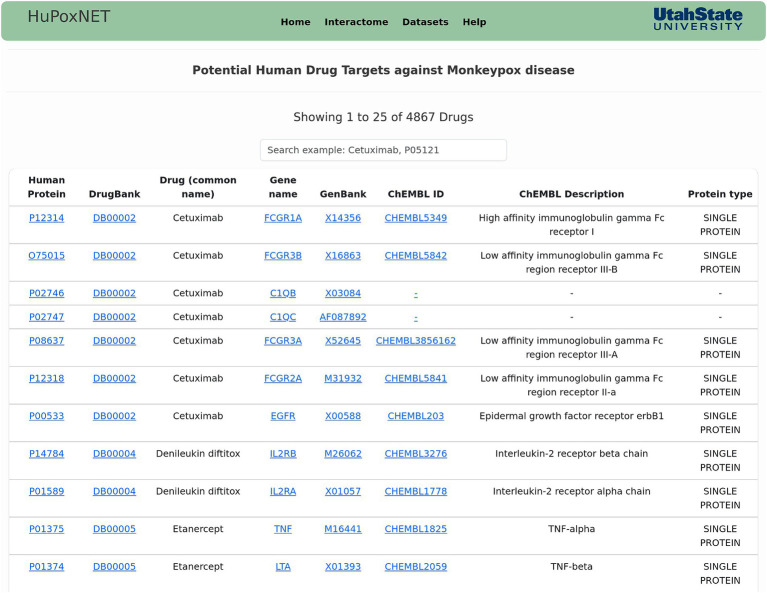
Snapshot of the “Drug Targets” page of HuPoxNET. This page contains the potential human drug targets against monkeypox disease, along with their respective gene names. The drug targets have been further linked to ChEMBL database for enhanced analysis.

Gene ontology provide meaningful annotations of the proteins, and are categorized into 3 domains—cellular component, biological process, and molecular function (Harris et al., 2006). The database contains the GO annotations of the human and viral proteins, along with the description and evidence code of the GO term. KEGG pathways and their respective description have also been implemented in the database. Subcellular localization is essential to understand the function of a protein in the cell. In context of PPIs, the identification of protein localization will provide insights into the location where the host and pathogen proteins interact during viral infection and human defense responses, thus aiding in the identification of potential targets for drug development (Pan et al., 2021). Furthermore, the functional domains of the proteins and related information such as protein length, InterPro ID, domain ID, and description of the domain has been made available for human and MPXV proteins. The potential drug targets for human proteins can also be accessed on the database, which direct to DrugBank for additional information on the drugs. For robust analysis of potential human proteins, these drug targets have been further linked to ChEMBL database^7^ (Figure 5), which is a comprehensive repository of bioactive drug-like molecules and contains information regarding their biological activities, mechanisms of action, and pharmacological properties (Gaulton et al., 2012).

The information from the above-mentioned protein functional annotations, wherever possible, has been linked to different biological databases. Furthermore, a search bar has been implemented on all the pages that allows the user to query a specific keyword such as kinase, ATP, etc. This makes the database highly efficient and easy to search information.

### 3.3 Application/case study

To increase reliability of the predicted interactions, we identified the validated drugs against monkeypox disease in the literature and searched those in the predicted interactome. Several studies have reported different drugs that were administered (orally, intravenous, or cutaneous) to the patients infected with MPXV, resulting in complete recovery from the disease. These Food and Drug Administration (FDA)-approved antiviral drugs include brincidofovir (Adler et al., 2022), tecovirimat (Almehmadi et al., 2022; Frenois-Veyrat et al., 2022; Matias et al., 2022), cidofovir (Tarín-Vicente et al., 2022; Srivastava et al., 2023), ribavirin (Baker et al., 2003) etc. that are used to treat poxviruses (Ortiz-Saavedra et al., 2022). Another study reported tigecycline as a therapeutic drug against MPXV infection by implementing structure-based virtual screening and molecular simulation (Alandijany et al., 2023). We also identified potential human targets for the above-mentioned drugs (Table 1).

**Table 1:** Human proteins and their potential drugs against monkeypox disease.

Human protein	Drug (common) name	Drug Bank ID
P16502	Ribavirin	DB00811
P26676	Ribavirin	DB00811
P12823	Ribavirin	DB00811
P33815	Tecovirimat	DB12020
P08546	Cidofovir	DB00369
P0AG59	Tigecycline	DB00560
P0A7S9	Tigecycline	DB00560
P0A7U3	Tigecycline	DB00560

Furthermore, we randomly selected a protein interaction pair (“Q92934-AAY97628.1”) from the predicted interactions in HuPoxNET and searched for the respective human and viral protein annotations in the database. The functional annotation information revealed that the human protein “Q92934” is involved in significant cellular processes including, apoptotic process (GO:0006915), positive regulation of apoptotic process (GO:0043065), mitochondrion (GO:0005739), BAD-BCL-2 complex (GO:0097138), etc. and molecular pathways such as apoptosis (map04210), PI3K-Akt signaling pathway (map04151), chemokine signaling pathway (map04062), and many others. This protein was also found to be localized in mitochondria. Furthermore, the protein “Q92934” was identified to be a pro-apoptotic member of Bcl-2 protein family (InterPro ID: IPR018868). Various studies have reported the regulation of apoptosis in mitochondria, which is tightly controlled by Bcl-2 protein family and its sub-domains such as Bcl-2 homology domain 3 (BH3) (Barber, 2001; Taylor et al., 2006). This results in programmed cell death of infected cells, thus interrupting viral DNA replication and subsequent release of progeny virus (Thomson, 2001). The process of apoptosis is also reported to be significantly regulated by PI3K-Akt signaling pathway and chemokine signaling pathway that are crucially involved in immune responses during viral infection (Vlahakis et al., 2002; Diehl and Schaal, 2013). The drug target for this human protein was found to be “Navitoclax” (DrugBank ID: DB12340), which is reported to be a high affinity inhibitor of Bcl-2 (Wilson et al., 2010). The ChEMBL ID associated with this protein was “CHEMBL5169266” (BCL2/BAD), which is a “protein–protein interaction” type molecule. Further analysis using ChEMBL database revealed that this human protein also interacts with a vaccinia virus protein (UniProt ID: P68451), which belongs to same genus (Orthopoxvirus) as that of monkeypox virus (Manenti et al., 2023).

The interacting viral protein “AAY97628.1” was involved in biological processes such as suppression by virus of host apoptotic process (GO:0019050), and regulation of apoptotic process (GO:0042981). This protein was also found to contain a VAC_F1L type conserved protein and apoptosis regulator M11L like protein. A study in the past showed that the proteins M11L (myxoma virus protein) and F1L (vaccinia virus protein) are localized in mitochondria and play a role in inhibiting host mitochondrial apoptosis by mimicking the function of Bcl-2 protein and blocking pro-apoptotic signals (Everett et al., 2000; Stewart et al., 2005; Douglas et al., 2007). The protein annotations of the human and viral proteins in the above-mentioned interaction pair make it a candidate pair for further analysis and experimental validation of the interaction. Additionally, this shows the reliability of the predicted interactions using the interactome tool and annotations of HuPoxNET. We believe many more potential interaction pairs exist that can be exploited to gain in-depth knowledge of the molecular mechanisms active during viral infection.

## Summary

In view of recent outbreak of monkeypox disease in humans, we have developed a user-friendly, comprehensive protein interaction database known as “HuPoxNET” (https://kaabil.net/hupoxnet/) that contains information about the protein–protein interactions involved in the disease, caused by various strains of monkeypox virus. Protein datasets of 22 diverse strains of monkeypox virus were implemented. Computational models with high accuracy were designed for PPIs prediction. The database will serve as a centralized repository for human-MPXV PPIs and the functional annotations of the human and viral proteins involved in the interactions. It is noteworthy here that our database does not contain machine learning-based (e.g., deep learning) predictions due to the limited availability of relevant dataset for training efficient models, which can lead to reduced performance of the model, thus affecting accuracy and reliability of predicted interactions. In the future versions of HuPoxNET, the database will be enhanced with more biological information of the proteins, and with a more advanced user interface and optimized performance for results retrieval. We also plan to incorporate and improve deep learning-based models as more training data becomes available.

## Data availability statement

HuPoxNET is available at: https://kaabil.net/hupoxnet/. All the datasets implemented on the database are available on: https://kaabil.net/hupoxnet/datasets/.

## Author contributions

Rkat: Data curation, Methodology, Formal analysis, Software, Writing – original draft, Visualization. ND: Software, Validation, Writing – review & editing. RKau: Conceptualization, Funding acquisition, Project administration, Resources, Supervision, Visualization, Writing – review & editing.

## Footnotes

^1^ https://www.cdc.gov/poxvirus/mpox/response/2022/index.html

^2^ https://www.uniprot.org/

^3^ https://www.ncbi.nlm.nih.gov/

^4^ https://useast.ensembl.org/index.html

^5^ https://www.proteinatlas.org/

^6^ https://go.drugbank.com/

^7^ https://www.ebi.ac.uk/chembl/
